# Preliminary  Validation of a Colorimetric Loop-Mediated Isothermal Amplification (c-LAMP) Assay for Detection of *Pythium insidiosum* in Clinical Specimens

**DOI:** 10.3390/jof12050351

**Published:** 2026-05-09

**Authors:** Thanawat Sridapan, Chalisa Jaturapaktrarak, Thidarat Rujirawat, Wilasinee Konsue, Pattarana Sae-Chew, Chompoonek Yurayart, Theerapong Krajaejun

**Affiliations:** 1 Department of Pathology, Faculty of Medicine, Ramathibodi Hospital, Mahidol University, Bangkok 10400, Thailand; 2 Offices of Health Science Research, Faculty of Medicine, Ramathibodi Hospital, Mahidol University, Bangkok 10400, Thailand; chalisa.jat@mahidol.edu (C.J.); thidarat.ruj@mahidol.ac.th (T.R.);; 3 Department of Microbiology and Immunology, Faculty of Veterinary Medicine, Kasetsart University, Bangkok 10900, Thailand; fvetcny@ku.ac.th

**Keywords:** *Pythium insidiosum*, pythiosis, diagnosis, LAMP, hydroxynaphthol blue

## Abstract

Pythiosis is an emerging infectious disease caused by the oomycete *Pythium insidiosum*, affecting humans and animals primarily in subtropical and tropical regions. The pathogen is commonly found in swampy environments, and exposure can lead to diverse clinical manifestations. In humans, ocular and vascular infections predominate, whereas in animals, cutaneous/subcutaneous or gastrointestinal disease is more common. Medical therapy is frequently ineffective, and many patients require extensive surgical intervention. Advanced cases may progress to fatal outcomes. Therefore, early and accurate detection is critical for improving clinical management. This study evaluated a colorimetric loop-mediated isothermal amplification (c-LAMP) assay compared with an established multiplex PCR (m-PCR) assay for the detection of *P. insidiosum* in clinical specimens from animals with and without pythiosis. When tested on 47 frozen tissue samples, c-LAMP demonstrated superior diagnostic performance, with markedly greater sensitivity (83.9% vs. 41.9%), higher accuracy (78.7% vs. 61.7%), and a shorter turnaround time (65 vs. 180 min). However, c-LAMP yielded five false-positive results, likely due to nonspecific amplification or contamination. Improved sample-handling practices increased the specificity from 68.8% to 93.8%. In contrast, m-PCR showed perfect specificity (100.0%) but substantially lower sensitivity, resulting in a high false-negative rate. In conclusion, these preliminary findings suggest that c-LAMP is a promising rapid screening tool for suspected pythiosis, particularly in resource-limited settings. Nevertheless, confirmatory testing remains necessary for positive or equivocal c-LAMP results.

## 1. Introduction

Pythiosis is an emerging infectious disease that affects both humans and various animals, caused by the fungal-like oomycete organism *Pythium insidiosum* [1]. It remains a significant global health threat, particularly in subtropical and tropical regions [2]. Globally, 94.3% of human cases have been reported in Thailand and India, while animal cases (horses, cattle, and dogs) are most prevalent in the U.S.A. and Brazil (79.2%), often associated with water exposure, as this pathogen is commonly found in agricultural swamps and stagnant freshwater environments [1,2]. The clinical manifestations of pythiosis can vary considerably [3]. In humans, the most common forms include ocular and vascular infections, while cutaneous/subcutaneous and gastrointestinal infections are more frequently observed in animals [2]. Unfortunately, medical treatments for pythiosis have often been ineffective. Consequently, many affected patients require surgical removal of the infected organ (such as a leg, eye, or intestine) to manage the infection, and in some cases, patients with advanced disease may ultimately succumb to uncontrolled infections [4,5]. To enhance patient outcomes, early and accurate diagnosis is essential for timely intervention and improved clinical results [5,6].

Traditional culture isolation is considered the gold standard for diagnosing pythiosis [1,6]. However, this method has limited sensitivity and is labor-intensive. To address these issues, immunological assays like ELISA and immunochromatographic tests have been developed [6,7]. Although immunoassays offer more accessible options and utilize simple devices for the rapid detection of anti-*P. insidiosum* antibodies, their effectiveness has raised concerns regarding specificity and the risk of cross-reactivity with other infections [8,9,10]. In recent years, nucleic acid amplification tests (NAATs), including PCR [11,12], nested PCR [13,14,15,16,17], multiplex PCR (m-PCR) [18,19], and real-time PCR [20,21], have gained popularity for their high sensitivity and specificity in directly detecting *P. insidiosum* [6,22]. However, a significant drawback of NAAT is its requirement for expensive equipment and skilled laboratory personnel [23]. Therefore, a simple and cost-effective NAAT could serve as a valuable alternative for detecting *P. insidiosum*, especially in resource-limited settings [22].

Loop-mediated isothermal amplification (LAMP), developed by Notomi et al., [24] was designed to address the limitations of NAAT [25,26]. The LAMP reaction utilizes four to six specially designed primers along with *Bst* DNA polymerase, which has auto-cycling strand-displacement activity. This combination enables the amplification of target DNA within an hour at a consistent temperature of 60–65 °C [24,26,27,28]. One significant advantage of the LAMP assay is that it requires only basic heating equipment, such as a water bath or heating block, rather than an expensive thermal cycler and electrophoresis apparatus used in PCR-based assays, making it a relatively cost-effective option for resource-limited settings [22,24,26,27,28]. The results of the assay can be observed instantly by the naked eye via colorimetric detection, which results in a color change [25,26,29]. Recently, we successfully developed a colorimetric LAMP assay (c-LAMP) using the metal indicator hydroxy naphthol blue (HNB) to facilitate straightforward readout in a closed-tube endpoint detection system [30,31]. This assay demonstrated 100.0% sensitivity, 95.7% specificity, and 97.5% accuracy in detecting DNA samples extracted from various strains of *P. insidiosum* [31]. This study aims to evaluate the effectiveness of the *P. insidiosum*-specific c-LAMP using clinical specimens. The results could enhance confidence in the clinical application of c-LAMP for diagnosing pythiosis.

## 2. Materials and Methods

### 2.1. Clinical Samples and DNA Preparation

All available frozen tissue samples (*n* = 47) were obtained from the Department of Microbiology and Immunology, Faculty of Veterinary Medicine, Kasetsart University, Bangkok, Thailand (Table 1). These comprised samples from 23 dogs, 22 horses, one cat, and one bird, including 31 from animals diagnosed with pythiosis and 16 from animals without pythiosis, which served as controls. The affected tissues comprised cutaneous/subcutaneous lesions (chronic wounds, kunkers, granulomas, and nodules), gastrointestinal masses (ileocecal, duodenal, gastric, jejunal, large intestinal, esophageal, and perianal), hepatopancreatic involvement (liver and pancreatic granulomas), respiratory lesions (nasal and air sac masses), musculoskeletal and hoof tissues (limbs, pelvis, and hoof), and other sites, including the lip, cheek, spleen, kidney, brain, bone marrow, and genital tissues. The clinical diagnoses of these animals were confirmed through culture isolation or routine molecular assays (PCR and sequence homology analysis [32]. The control samples were from infections caused by various fungal species (*Basidiobolus ranarum*, *Aspergillus flavus*, *Aspergillus fumigatus*, *Cryptococcus laurentii*, and *Lasiodiplodia theobromae*) or from cases in which culture or molecular assays did not detect any organism (Table 1). The inclusion of these non-*Pythium* organisms and negative samples was intended to evaluate assay specificity under realistic diagnostic conditions, where distinguishing *P. insidiosum* from similar fungi or negative cases is critical. For DNA extraction, the tissue samples were ground in liquid nitrogen using a mortar and pestle, following established protocols [33,34]. The extracted DNA was dissolved in 50 µL of Tris-HCl (pH 8.5) and stored at −20 °C until further analysis. All samples were processed blindly and tested for the presence of *P. insidiosum* DNA using both the c-LAMP and m-PCR methods [18,31,32].

### 2.2. c-LAMP Assay

The c-LAMP primer set used in this study was adopted from our previous work [31]. These primers were evaluated for self-complementarity and potential secondary structure formation (e.g., hairpins or primer-dimers) using the default settings of Primer Explorer V5 (http://primerexplorer.jp/lampv5e/index.html; accessed on 8 July 2024). The c-LAMP assay was performed as previously described [31]. In brief, each 25 μL reaction included the following components: 1× ThermoPol buffer, 6 mM MgSO_4_, 1.4 mM dNTPs, 8 U of *Bst* DNA polymerase large fragment (New England Biolabs Inc., Ipswich, MA, USA), 0.4 M betaine (Sigma-Aldrich, St. Louis, MO, USA), and an ITS primer mix consisting of 0.2 μM F3 (5′-GGCAGAATGTGAGGTGTCTC-3′) and B3 (5′-GGAAACAACACCCCGTCAG-3′), 1.6 μM FIP (5′-ACAGATCACTGCGTTCGAGCAT-TTTT-GGAGATAGCACGAGTCCCT-3′) and BIP (5′-TCAGATTGCTTTGCGCTGGTGG-TTTT-CCGAAGCCTAACATACCGC-3′), and 0.4 μM LF (5′-GACACAAGAGAGATCAACGTACATT-3′) and LB (5′-AGGACATTAAGGAGATGACCTCTAT-3′) (Bio Basic Inc., Markham, ON, Canada). Additionally, 0.12 mM HNB solution [30] (3 mM, Loba Chemie, Mumbai, Maharashtra, India) and 2 μL of template DNA were added to the reaction. The reaction was conducted in a thermal cycler at 65 °C for 60 min. A colorimetric change in the metal indicator HNB from violet to sky blue indicated a positive result, as assessed by the naked eye. c-LAMP products were further confirmed using agarose gel electrophoresis, stained with SERVA DNA stain G (SERVA Electrophoresis GmbH, Heidelberg, Germany), and visualized under a UV transilluminator (Bio-Rad, Hercules, CA, USA). The presence of a step ladder band pattern indicates *P. insidiosum*. To reduce the likelihood of false-negative results, all DNA samples were quantified by spectrophotometry to ensure sufficient template was present and tested in parallel by both c-LAMP and m-PCR (see below). Each c-LAMP run also included appropriate positive (DNA samples extracted from *P. insidiosum* strains CBS573.85, Pi-S, and MCC13) and negative (nuclease-free water; no-template or NTC) controls.

### 2.3. m-PCR Assay

The m-PCR analysis was performed according to the previously reported protocol [18,32]. The reaction mixture had a total volume of 25 µL and contained 1 × *Taq* buffer with KCl, 2 mM MgCl_2_, 0.2 mM dNTPs, and 0.75 units of *Taq* DNA polymerase (Thermo Fisher Scientific, Waltham, MA, USA). Additionally, 2 μL of template DNA was included, along with primers: 0.12 μM of the forward primer ITS1 (5′-TCCGTAGGTGAACCTGCGG-3′) and 0.07 μM each of the reverse primers R1 (5′-CCTCACATTCTGCCATCTCG-3′), R2 (5′-ATACCGCCAATAGAGGTCAT-3′), and R3 (5′-TTACCCGAAGGCGTCAAAGA-3′). Nuclease-free water served as NTC. The amplification reaction was performed in a Mastercycler Nexus Gradient thermocycler (Eppendorf, Hamburg, Germany) using the following settings: 95 °C for 5 min; 30 cycles of 95 °C for 30 s, 59 °C for 30 s, and 72 °C for 45 s; followed by a final extension step at 72 °C for 10 min. The resulting m-PCR products were analyzed by agarose gel electrophoresis, as described above for the c-LAMP products. The presence of 490 bp and 660 bp amplicons (two bands), a 660 bp amplicon, or an 800 bp amplicon indicates clade-I, clade-II, and clade-III *P. insidiosum* strains, respectively.

### 2.4. Statistical Analysis

The diagnostic performance of the c-LAMP and m-PCR assays was evaluated for sensitivity, specificity, positive predictive value (PPV), negative predictive value (NPV), and accuracy. This analysis was conducted using the MedCalc statistical software (https://www.medcalc.org/calc/diagnostic_test.php; accessed on 23 November 2025). All estimates were reported with 95% confidence intervals (CI).

### 2.5. Sequence Analysis for Similarities and Mismatches

The rDNA ITS sequence of *P. insidiosum* was searched against the NCBI database (https://blast.ncbi.nlm.nih.gov/Blast.cgi; accessed on 22 October 2024) using blastn to identify homologous sequences in other organisms. This sequence was also aligned with those from other fungal species that produced false-positive c-LAMP reactions, utilizing the Clustal W algorithm within BioEdit version 7.2.5 [35] to examine similarities and mismatches. Additionally, all c-LAMP primers were assessed for potential cross-annealing with the target rDNA ITS sequences from other fungi.

## 3. Results and Discussion

### 3.1. Diagnostic Performance of c-LAMP Compared with m-PCR

As a complementary approach, we selected another molecular method to strengthen the evaluation of the c-LAMP assay. Quantitative PCR (qPCR), also known as real-time PCR, is a powerful diagnostic tool with high sensitivity and quantitative capability [20,21]. However, it relies on advanced equipment, specialized reagents, and fluorescent dyes, which may limit its applicability in resource-limited settings. In this study, we therefore used m-PCR [18], which is routinely used in our laboratory to detect *P. insidiosum*, as the reference method for comparison. Future studies incorporating qPCR, particularly in well-equipped laboratories, alongside a composite reference standard (e.g., culture, histopathology, sequencing, and clinical diagnosis), could further strengthen confidence in the diagnostic performance of c-LAMP.

We evaluated the diagnostic performance of the c-LAMP assay using clinical specimens and compared its results with those obtained from the established m-PCR method [18]. A total of 47 frozen tissue samples were examined, comprising 31 samples from animals diagnosed with pythiosis and 16 non-pythiosis controls (Table 1). The control group included tissues from animals infected with other fungal pathogens (such as *Aspergillus*, *Basidiobolus*, and *Cryptococcus* species) and samples with negative culture results. Genomic DNA was extracted from all samples, blinded, and randomly assigned identification numbers before testing (Table 1). The examiner was unaware of the clinical status of each specimen.

**Figure 1:**
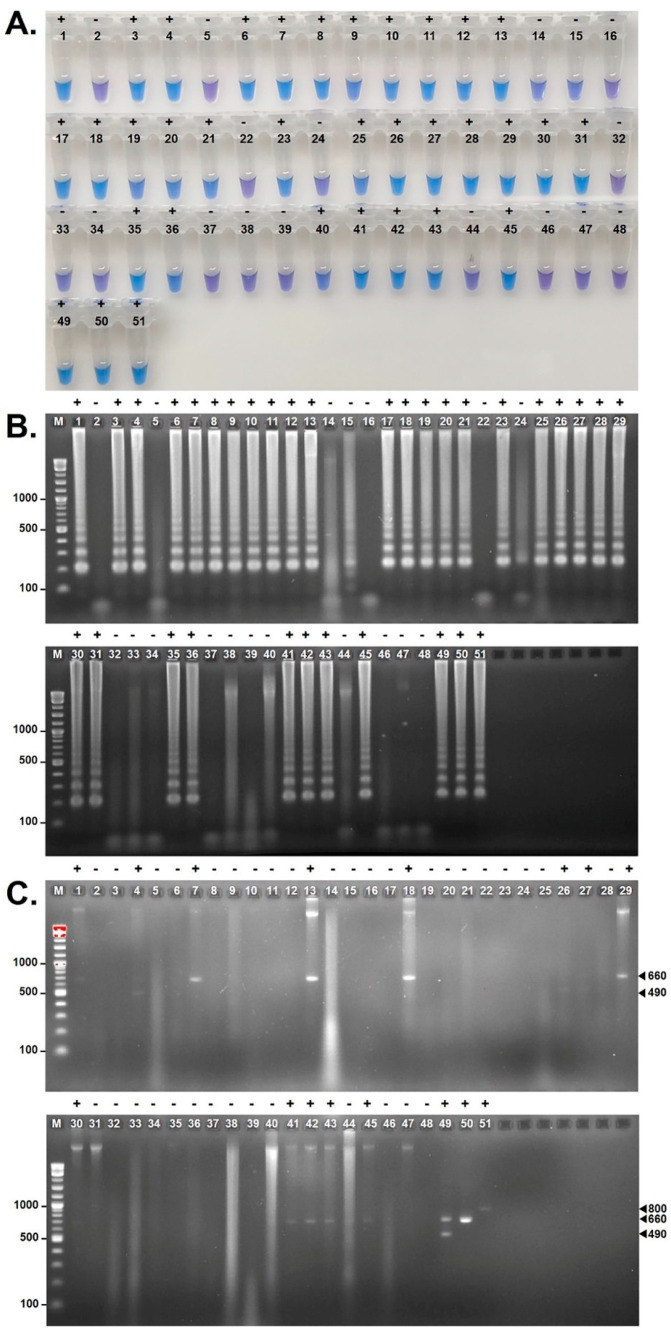
Evaluation of the c-LAMP and m-PCR assays for detecting *Pythium insidiosum* in blinded clinical samples. Results are shown as: (**A**) Colorimetric outcomes of the c-LAMP assay using the metal indicator hydroxy naphthol blue (HNB), where sky blue indicates a positive (+) reaction and violet indicates a negative (−) reaction; (**B**) Agarose gel electrophoresis of c-LAMP products, where the characteristic step-ladder banding pattern indicates *P. insidiosum*; and (**C**) Agarose gel electrophoresis of m-PCR products, in which 490/660 bp (2 bands), 660 bp, and 800 bp amplicons correspond to clade I, clade II, and clade III *P. insidiosum* strains, respectively. Numeric labels represent clinical tissue samples 1–47 (see Table 1). Sample 48 is the no-template control (NTC). Samples 49–51 represent positive control DNAs extracted from *P. insidiosum* strains CBS573.85 (clade I), Pi-S (clade II), and MCC13 (clade III), respectively. “M” indicates DNA ladder markers.

The c-LAMP assay demonstrated strong diagnostic performance, correctly identifying 26 of 31 pythiosis cases, missing 5, and yielding 5 false-positive results among the controls (Figure 1). This corresponded to a sensitivity of 83.9% [95% CI: 66.3–94.6%] and a specificity of 68.8% [95% CI: 41.3–89.0%]. The PPV was 83.9% [95% CI: 71.2–91.6%], indicating that most positive reactions reflected true *P. insidiosum* infections, while the NPV was 68.8% [95% CI: 48.0–84.0%], showing moderate reliability for ruling out pythiosis. In contrast, the m-PCR assay detected only 13 pythiosis cases and missed 18, but produced no false positives among the controls (Figure 1). Its sensitivity was 41.9% [95% CI: 24.6–60.9%], and its specificity was 100.0% [95% CI: 79.4–100.0%]. The PPV was 100.0% [95% CI: 75.3–100.0%], confirming that all positive m-PCR results were true positives, whereas the NPV was low at 47.1% [95% CI: 39.7–54.5%] due to the high false-negative rate.

The marked difference in sensitivity between c-LAMP and m-PCR warrants particular attention, as missed diagnoses represent a critical limitation in the clinical detection of pythiosis. In this context, the relatively low sensitivity of the m-PCR assay, reflected by its high false-negative rate, likely constrained its diagnostic utility when applied to clinical specimens. The higher sensitivity of c-LAMP observed in the present study is consistent with our previous work using pure culture samples, in which c-LAMP also outperformed m-PCR in diagnostic sensitivity [31]. This difference can be explained by the substantially lower limit of detection (LOD) of the c-LAMP assay, which can detect as little as 1 × 10^−5^ ng of *P. insidiosum* DNA, whereas the m-PCR assay has a detection limit of 0.1 ng. This lower detection threshold is a particular advantage for clinical samples, in which low pathogen burden or DNA degradation may occur, and likely contributed to the improved sensitivity of c-LAMP observed in this study.

These performance characteristics were reflected in the overall accuracy (proportion of correct results) of each method: c-LAMP achieved an accuracy of 78.7% [95% CI: 64.3–89.3], substantially higher than the 61.7% [95% CI: 46.4–75.5] observed for m-PCR. Additionally, c-LAMP required considerably less laboratory time, with a total reaction time of 65 min compared with 180 min for m-PCR, which involved longer amplification steps and agarose-gel electrophoresis.

### 3.2. Causes of False-Positive and False-Negative Results in c-LAMP

Previous studies evaluating LAMP for the detection of *P. insidiosum* have reported high analytical sensitivity and specificity, particularly when assays were applied to DNA extracted from pure fungal cultures. Early LAMP protocols employed a 4-primer set and required post-amplification open-tube processing, such as gel electrophoresis, which increased the risk of DNA carryover contamination [32]. Subsequently, a c-LAMP assay incorporating a 6-primer set and the metal indicator hydroxy naphthol blue (HNB) was developed to enable closed-tube endpoint detection and improve amplification efficiency, and this approach demonstrated excellent diagnostic performance when evaluated using culture-derived DNA [31]. In contrast, data on the performance of c-LAMP using DNA extracted directly from infected tissue or other clinical specimens have remained limited. In the present study, application of c-LAMP to clinical samples resulted in reduced diagnostic accuracy, with false-positive results in 5 of 16 control samples and false-negative results in 5 of 31 confirmed pythiosis samples, yielding a sensitivity of 83.9% and a specificity of 68.8%. This performance was notably lower than that obtained using culture-derived DNA in our previous study (100.0% sensitivity and 95.7% specificity) [31]. These findings suggest that assay performance is influenced by the nature and quality of the DNA template.

Five false-negative results were observed with the c-LAMP assay, and several factors may account for these findings. Although c-LAMP demonstrated excellent analytical sensitivity (detecting as little as 1 × 10^−5^ ng of *P. insidiosum* DNA [31]), some clinical specimens likely contained DNA quantities below this threshold, particularly when the fungal burden within tissues was low or unevenly distributed. Additionally, partial degradation or loss of *P. insidiosum* DNA during extraction from frozen tissue samples could reduce the availability of intact target sequences needed for efficient primer binding and amplification. Both issues would compromise assay sensitivity. These limitations align with our diagnostic results: c-LAMP correctly identified 26 of 31 confirmed pythiosis cases but failed to detect 5, yielding a sensitivity of 83.9%, which remained substantially higher than that of m-PCR (41.9%). The false-negative rate could likely be reduced, thus improving the sensitivity of both c-LAMP and m-PCR, by increasing the number of sampling sites from affected lesions, repeating testing with newly collected tissue, and optimizing DNA extraction procedures to minimize DNA loss.

**Figure 2:**
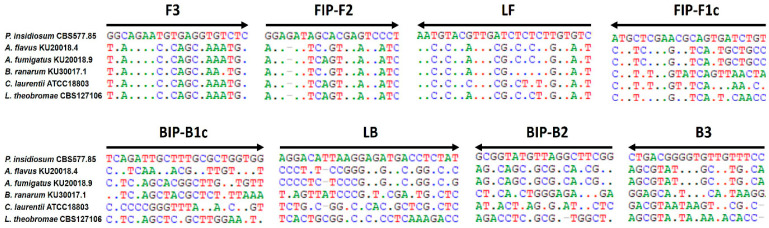
Sequence alignment of the c-LAMP primers and rDNA ITS sequences from *Pythium insidiosum* and other fungal organisms. The c-LAMP primer set includes F3, B3, FIP (comprising the FIP-F2 and FIP-F1c regions), BIP (comprising the BIP-B1c and BIP-B2 regions), LF, and LB. Arrows indicate the primers (5′→3′ direction) and show their corresponding annealing sites within the target rDNA ITS sequences of *P. insidiosum* (strain CBS 577.85) and five fungal species [i.e., *Aspergillus flavus* (KU20018.9), *Aspergillus fumigatus* (KU20018.9), *Basidiobolus ranarum* (KU30017.1), *Cryptococcus laurentii* (ATCC 18803), and *Lasiodiplodia theobromae* (CBS 127106)]. A, T, C, and G represent nucleotides, and dots indicate positions with identical nucleotides among the aligned sequences.

The five false-positive results obtained with c-LAMP are likely due to inherent characteristics of the LAMP technique. LAMP is an isothermal amplification method that rapidly generates extremely large quantities of DNA, making it more susceptible to amplification from trace contaminants or non-specific primer interactions [36,37,38]. Although the false-positive samples were confirmed by culture and rDNA ITS sequence homology analysis to contain other fungal organisms (i.e., *Aspergillus flavus*, *A. fumigatus*, *Basidiobolus ranarum*, *Cryptococcus laurentii*, and *Lasiodiplodia theobromae*; Table 1), comprehensive in silico analyses (including blastn screening of the 233 bp target region against the NCBI database (https://blast.ncbi.nlm.nih.gov/Blast.cgi; accessed on 22 October 2024) and primer-binding assessment using BioEdit version 7.2.5 [35]) showed no detectable sequence similarity between these organisms and the c-LAMP primer targets (Figure 2). These results strongly indicate that the false positives were not due to true genetic cross-reactivity but instead arose from accidental cross-contamination during sample handling or from the intrinsic high sensitivity of LAMP to minute quantities of contaminating nucleic acids [36,39]. In contrast, m-PCR has substantially lower analytical sensitivity and is therefore less prone to false-positive amplification [31].

Sample tracing indicated that the aliquoted tubes corresponding to these false-positive samples (Table 1) had been reused and shared across multiple experiments, increasing the likelihood of cross-contamination. To investigate this possibility, we repeated the c-LAMP assay using new aliquots containing DNA extracted from the same five tissue samples. Only Sample 9 (derived from an animal with *Basidiobolus* infection) consistently produced a false-positive result, underscoring both the potential impact of sample contamination and the extremely high analytical sensitivity of c-LAMP. Importantly, eliminating such sources of interference could markedly reduce the false-positive rate and improve the detection specificity of c-LAMP from 68.8% to 93.8%.

### 3.3. Artifacts in c-LAMP Result Interpretation

Interpreting the color change from violet (negative) to sky blue (positive) in c-LAMP requires careful consideration. In one instance, Sample 40, which was particularly viscous, exhibited an immediate transition to sky blue upon sample addition, even before amplification. This premature color shift likely resulted from a high concentration of extracted DNA, which has a strong negative charge due to its phosphate backbone that can bind tightly to Mg^2+^ ions. An excess of template DNA may chelate these Mg^2+^ ions, reducing their availability and leading to the early color change. To mitigate this artifact, diluting the viscous sample 10-fold with sterile distilled water proved effective. These findings highlight the importance of assessing sample viscosity and pre-treating problematic extracts prior to c-LAMP analysis. Conversely, clinical samples containing very low quantities of *P. insidiosum* DNA (near the analytical detection limit) may produce only a subtle color shift. This occurs because limited amplification yields only small amounts of magnesium pyrophosphate, resulting in an insufficient reduction in Mg^2+^ ions to generate a distinct HNB-based color change [40]. Such borderline reactions can be difficult to interpret visually.

To improve reliability in clinical settings, we recommend diluting viscous DNA extracts 10-fold before performing c-LAMP, visually confirming that all reaction tubes remain violet prior to amplification, and verifying any positive or ambiguous colorimetric results using agarose gel electrophoresis. Together, these measures reduce misinterpretation and enhance the overall diagnostic accuracy of the c-LAMP assay. Because c-LAMP relies on visual interpretation of color change, inter-observer variability may occur. To address this limitation, objective readout methods should be explored. Potential approaches include OD measurement and artificial intelligence (AI)-assisted image analysis of reaction tubes. These strategies could provide more consistent and reproducible interpretation, thereby reducing subjectivity and improving overall result reliability.

### 3.4. Clinical Implications of c-LAMP

In this preliminary study, we evaluated the diagnostic performance of a c-LAMP assay for detecting *P. insidiosum* in clinical tissue samples and compared it with m-PCR. Although the c-LAMP assay provided a rapid turnaround time and high analytical sensitivity, an initially high false-positive rate was observed, resulting in a specificity of 68.8%. This represents a clinically relevant limitation that restricts the assay’s use as a standalone diagnostic test. False-positive results are of particular concern in pythiosis, as misdiagnosis may lead to unnecessary invasive surgical procedures or prolonged systemic antifungal therapy, both associated with substantial risk and potential long-term morbidity.

Analysis of discordant results suggested that false positives were largely attributable to sample contamination associated with pre-analytical handling practices, including reuse of aliquoted material across experiments, as well as the inherently high sensitivity of c-LAMP. Following implementation of stricter pre-analytical controls and improved sample handling, assay specificity increased from 68.8% to 93.8% under optimized conditions, highlighting the strong influence of workflow management and laboratory practice on test performance. Nevertheless, the potential for false-positive results remains a major methodological limitation, underscoring the need for a rigorous contamination-prevention workflow to ensure reliable diagnostic validation of highly sensitive amplification assays.

Consequently, confirmatory testing using an independent molecular method may be required for positive or ambiguous c-LAMP results. While this reduces some advantages of c-LAMP, including rapid turnaround time and low cost, it is necessary to ensure diagnostic accuracy and patient safety. Rather than serving as a definitive diagnostic tool, c-LAMP is therefore best positioned as a rapid screening assay in the generally low-prevalence setting of pythiosis, enabling rapid exclusion of disease in many suspected cases while reserving confirmatory testing (e.g., m-PCR) for a limited subset of samples.

## 4. Conclusions

In this preliminary study using 47 frozen tissue samples from affected animals, the c-LAMP assay showed higher overall diagnostic performance than m-PCR, achieving an accuracy of 78.7% compared with 61.7% for m-PCR. Although c-LAMP showed substantially higher sensitivity (83.9% vs. 41.9%) and a markedly shorter turnaround time (65 vs. 180 min), an elevated false-positive rate under suboptimal handling conditions limits its use as a standalone diagnostic assay and represents a potentially significant clinical concern given the serious consequences of misdiagnosing pythiosis. In this preliminary dataset, c-LAMP produced 5 false negatives and 5 false positives, with most false-positive results attributable to contamination or non-specific amplification. However, improved sample handling increased assay specificity from 68.8% to 93.8%, underscoring the importance of pre-analytical management. In contrast, m-PCR exhibited perfect specificity (100.0%) but poor sensitivity, resulting in a high false-negative rate and a low NPV (47.1%). While interpretation artifacts may complicate c-LAMP analysis in viscous or low-DNA samples, simple corrective measures can enhance diagnostic reliability. Based on these findings, when implemented with appropriate quality control and interpreted alongside clinical data, c-LAMP may be most appropriately applied as a rapid screening tool for suspected pythiosis, with confirmatory testing (e.g., m-PCR) considered for positive or ambiguous results. Nevertheless, further validation in larger, more diverse clinical cohorts, including human and animal fresh clinical specimens (as frozen tissue may influence DNA integrity and diagnostic performance), is required to support robust conclusions regarding its clinical utility.

## Figures and Tables

**Table 1:** List of blind clinical samples from the tissues of animals with (*n* = 31) or without (*n* = 16) pythiosis, used to evaluate the diagnostic performance of the c-LAMP and m-PCR assays.

Sample Number	Source Animal	Tissue Affected	Causative Organism *	c-LAMP Result	m-PCR Result
1	Horse	Chronic wound, kunkers	*Pythium insidiosum*	+	+
2	Dog	Liver, granuloma	*Pythium insidiosum*	−	−
3	Dog	Ileocecal mass	*Pythium insidiosum*	+	−
4	Dog	Chronic wound, granuloma	*Pythium insidiosum*	+	+
5	Horse	Lips, granuloma	*Basidiobolus ranarum*	−	−
6	Dog	Duodenum, granuloma	*Pythium insidiosum*	+	−
7	Horse	Heel bulb, kunkers	*Pythium insidiosum*	+	+
8	Dog	Gastric mass	*Pythium insidiosum*	+	−
9	Horse	Lips, granuloma	*Basidiobolus ranarum*	+	−
10	Dog	Pancreas, granuloma	*Pythium insidiosum*	+	−
11	Dog	Perianal mass	*Pythium insidiosum*	+	−
12	Dog	Ileocecal mass	*Pythium insidiosum*	+	−
13	Horse	Kunkers	*Pythium insidiosum*	+	+
14	Horse	Lips, granuloma	*Basidiobolus ranarum*	−	−
15	Dog	Ileocecal mass	*Pythium insidiosum*	−	−
16	Dog	Jejunum granuloma	*Pythium insidiosum*	−	−
17	Dog	Jejunum granuloma	*Pythium insidiosum*	+	−
18	Horse	Kunkers	*Pythium insidiosum*	+	+
19	Horse	Nasal mass	*Aspergillus flavus*	+	−
20	Horse	Cheek, mass	*Lasiodiplodia theobromae*	+	−
21	Bird	Air sacs, nodule	*Aspergillus fumigatus*	+	−
22	Dog	Esophageal mass	No organism identified	−	−
23	Cat	Generalized skin nodules	*Cryptococcus laurentii*	+	−
24	Dog	Inguinal mass	No organism identified	−	−
25	Dog	Anal granuloma	*Pythium insidiosum*	+	−
26	Dog	Esophageal granuloma	*Pythium insidiosum*	+	+
27	Dog	Hind limbs and pelvis granuloma	*Pythium insidiosum*	+	+
28	Horse	Distal limb tissue	*Pythium insidiosum*	+	−
29	Horse	Hoof tissue	*Pythium insidiosum*	+	+
30	Horse	Hoof tissue	*Pythium insidiosum*	+	+
31	Horse	Distal limb tissue	*Pythium insidiosum*	+	−
32	Dog	Spleen tissue	No organism identified	−	−
33	Dog	Kidney tissue	No organism identified	−	−
34	Dog	Brain tissue	No organism identified	−	−
35	Horse	Fetlock tissue	*Pythium insidiosum*	+	−
36	Dog	Large intestine granuloma	*Pythium insidiosum*	+	−
37	Dog	Stomach and large intestine granuloma	*Pythium insidiosum*	−	−
38	Dog	Bone marrow tissue	No organism identified	−	−
39	Dog	Liver tissue	No organism identified	−	−
40	Horse	Fetlock tissue	*Pythium insidiosum*	+	−
41	Horse	Hoof tissue	*Pythium insidiosum*	+	+
42	Horse	Distal limb tissue	*Pythium insidiosum*	+	+
43	Horse	Distal limb tissue	*Pythium insidiosum*	+	+
44	Horse	Fetlock tissue	*Pythium insidiosum*	−	−
45	Horse	Fetlock tissue	*Pythium insidiosum*	+	+
46	Horse	Penis granuloma	No organism identified	−	−
47	Horse	Lip mass	No organism identified	−	−

* Confirmed by culture isolation or rDNA ITS sequence homology analysis (+ Positive amplification reaction; − Negative amplification reaction). Non-*P. insidiosum* organisms and culture- or molecular assay-negative samples are included to assess assay specificity.

## Data Availability

The original contributions presented in this study are included in the article. Further inquiries can be directed to the corresponding author.
